# Dynamic Causal Modeling of Hippocampal Links within the Human Default Mode Network: Lateralization and Computational Stability of Effective Connections

**DOI:** 10.3389/fnhum.2016.00528

**Published:** 2016-10-25

**Authors:** Vadim Ushakov, Maksim G. Sharaev, Sergey I. Kartashov, Viktoria V. Zavyalova, Vitaliy M. Verkhlyutov, Boris M. Velichkovsky

**Affiliations:** ^1^National Research Centre “Kurchatov Institute”Moscow, Russia; ^2^Department of Cybernetics, National Research Nuclear University “MEPhI”Moscow, Russia; ^3^Higher School of Economics, National Research UniversityMoscow, Russia; ^4^Institute for Higher Nervous Activity and Neurophysiology, Russian Academy of SciencesMoscow, Russia; ^5^NBICS-Faculty, Moscow Institute of Physics and TechnologyMoscow, Russia; ^6^Faculty of Psychology, M.V. Lomonosov Moscow State UniversityMoscow, Russia; ^7^Center for Cognitive Programs and Technologies, Russian State University for the HumanitiesMoscow, Russia; ^8^Applied Cognitive Research, Department of Psychology, Technische Universitaet DresdenDresden, Germany

**Keywords:** effective connectivity, functional connectivity, default mode network, resting state networks, hippocampal asymmetry, DCM, Dynamic Causal Modeling fMRI

## Abstract

The purpose of this paper was to study causal relationships between left and right hippocampal regions (LHIP and RHIP, respectively) within the default mode network (DMN) as represented by its key structures: the medial prefrontal cortex (MPFC), posterior cingulate cortex (PCC), and the inferior parietal cortex of left (LIPC) and right (RIPC) hemispheres. Furthermore, we were interested in testing the stability of the connectivity patterns when adding or deleting regions of interest. The functional magnetic resonance imaging (fMRI) data from a group of 30 healthy right-handed subjects in the resting state were collected and a connectivity analysis was performed. To model the effective connectivity, we used the spectral Dynamic Causal Modeling (DCM). Three DCM analyses were completed. Two of them modeled interaction between five nodes that included four DMN key structures in addition to either LHIP or RHIP. The last DCM analysis modeled interactions between four nodes whereby one of the main DMN structures, PCC, was excluded from the analysis. The results of all DCM analyses indicated a high level of stability in the computational method: those parts of the winning models that included the key DMN structures demonstrated causal relations known from recent research. However, we discovered new results as well. First of all, we found a pronounced asymmetry in LHIP and RHIP connections. LHIP demonstrated a high involvement of DMN activity with preponderant information outflow to all other DMN regions. Causal interactions of LHIP were bidirectional only in the case of LIPC. On the contrary, RHIP was primarily affected by inputs from LIPC, RIPC, and LHIP without influencing these or other DMN key structures. For the first time, an inhibitory link was found from MPFC to LIPC, which may indicate the subjects’ effort to maintain a resting state. Functional connectivity data echoed these results, though they also showed links not reflected in the patterns of effective connectivity. We suggest that such lateralized architecture of hippocampal connections may be related to lateralization phenomena in verbal and spatial domains documented in human neurophysiology, neuropsychology, and neurolinguistics.

## Introduction

A set of functionally and structurally connected brain areas that are activated in a resting state and deactivated by external stimulation or cognitively effortful tasks have been identified as the default mode network (DMN) in several early papers (Gusnard et al., 2001; Gusnard and Raichle, 2001; Raichle et al., 2001). A number of hypotheses on the functionality of the DMN have been formulated mostly relating it to higher-order aspects of consciousness and cognition (Gusnard and Raichle, 2001; Gusnard et al., 2001; Schilbach et al., 2008).

The main part of the DMN has been identified in the medial prefrontal cortex (MPFC), posterior cingulate cortex (PCC), and the inferior parietal cortex (IPC) of both hemispheres (Buckner et al., 2008). Key DMN regions are well-connected structurally (Vogt et al., 2006; van den Heuvel et al., 2008, 2009; Greicius et al., 2009) and functionally (Greicius et al., 2003; Biswal et al., 2010). The MPFC and PCC are linked by powerful cingulate paths, which are identified by diffusion tensor imaging (DTI) (van den Heuvel et al., 2008). IPC is a functionally heterogeneous region involved in visual-spatial orientation, attention, memory, and math knowledge (Uddin et al., 2010). Paths connecting IPC and PPC were identified using a probabilistic approach of DTI (Khalsa et al., 2014; Tao et al., 2015). With respect to hippocampal formation (HF), rank correlations of activity also reveal the basic pattern of activation/deactivation characteristic of the DMN (Greicius et al., 2004; Vincent et al., 2006). Functional connectivity (FC) of both hippocampi has been analyzed in studies (Cui et al., 2015; Robinson et al., 2015) e.g., in a recent meta-analytic study by Robinson et al. (2016). However, causal relations of HF to other brain structures in the resting state have been investigated far less systematically than interactions between the key DMN regions.

An appropriate method to study these interactions is dynamic causal modeling (DCM), a Bayesian approach typically used to explain effective connectivity changes underlying task-related brain responses (Friston et al., 2003). The DCM estimates effective connectivity, which is the measure that mediates the influence one neuronal system exerts on another. DCM treats the brain as a ‘black box’ which receives input and generates output (Razi and Friston, 2016). To model the resting-state, a new version of DCM was introduced based on a deterministic model that generates predicted cross spectra, the spectral DCM (Friston et al., 2014). The resting-state fMRI signals convey fluctuations in the low-frequency band typically within 0.01–0.08 Hz (Biswal et al., 1995). To identify active regions of the DMN and corresponding time-series, the resting state is modeled using a generalized linear model containing a discrete cosine basis set with frequencies in the corresponding range, in addition to the individual nuisance regressor (Fransson, 2005; Kahan et al., 2014). After this, DCM models are specified in terms of nodes and possible connections between them. For example, in the present study, we consider two model sets with LHIP/RHIP inclusion together with the basic four DMN nodes, and one set with LHIP inclusion and PCC exclusion from the basic nodes. The best DCM model at the group level is then determined using the Bayesian model selection (BMS) procedure (Penny et al., 2004; Stephan et al., 2009).

The first work on the dynamic modeling of the causal links of DMN revealed that MPFC exerts greater effect on PCC than vice-versa, while bilateral IPC transmit information in the PCC and the MPFC (Di and Biswal, 2014). In addition, the authors found that the endogenous exposure may be greater in the right hemisphere than in the left. The introduction of full connectivity, taking into account inter-hemispheric connections between IPC, partially destroys the symmetry of these components but retains the main trend – the predominance of MPFC impact on PPC (Bastos-Leite et al., 2015). In a recent DCM analysis of causal relations between the HF, MPFC, the anterior cingulate cortex (ACC) and dorsolateral prefrontal cortex (DLPFC) Cui et al. (2015) showed slight asymmetry with a predominance of effects from MPFC to the right and left parts of HF, at least in healthy subjects. At the same time, the left MPFC seems to exert a stronger influence which is more apparent on the same side.

The present study has two related objectives. Firstly, it aims at localizing the hippocampal regions within the known structure of DMN causal connections, i.e., to examine effective connectivity between the four core DMN nodes and left and right parahippocampal regions estimating the coupling parameters. The second objective is to evaluate the model stability when adding or excluding regions of interest. For example, does the established connectivity pattern between sources change by addition of a new source, such as the left hippocampus? If not, then it could be possible to increase model complexity beginning with the model for well-known DMN sources by adding other candidate structures to participate in coupling. When estimating a more complex model, one could be confident that the previous connectivity pattern does not change much and all changes in the extended model are associated with the new source (region of interest). In our previous work (Sharaev et al., 2016), we found a stable coupling pattern between the four basic DMN nodes. Here, we aimed at developing the model to assess its parameter stability when adding or removing active regions. As in the previous work, endogenous fluctuations were explicitly modeled using Discrete Cosine Set and spectral DCM was applied to find effective connectivity patterns. To test only biologically plausible hypotheses on effective connectivity and to reduce the model space, we used structural connectivity data described by Tao et al. (2015).

## Materials and Methods

### Subjects

MRI data was obtained from 30 healthy subjects (10 males and 20 females, all right-handed without neurological symptoms), mean age 24 (range from 20 to 35 years). Consent from each participant was provided. Each participant was asked about their wakefulness during the study. Those who fell asleep in scanner were excluded from the study. Permission to undertake this experiment was granted by the Ethics Committee of the Institute of Higher Nervous Activity and Neurophysiology of the Russian Academy of Sciences.

### Scanning Parameters

The MRI data was acquired using a SIEMENS Magnetom Verio 3 Tesla. The T1- weighted sagittal three-dimensional magnetization-prepared rapid gradient echo sequence was acquired with the following imaging parameters: 176 slices, TR = 1900 ms, TE = 2.19 ms, slice thickness = 1 mm, flip angle = 9°, inversion time = 900 ms, and FOV = 250 mm × 218 mm. FMRI data was acquired with the following parameters: 30 slices, TR = 2000 ms, TE = 25 ms, slice thickness = 3 mm, flip angle = 90°, and FOV = 192 mm × 192 mm. Also, we acquired data which contain the options for reducing the spatial distortion of EPI images. For spectral DCM, (root) mean square error decreases as the number of time points increases, based on results from (Razi et al., 2015). Therefore, we decided to acquire 1000 time points (with a repetition time of 2 s), resulting in approximately 35 min of scanning.

### Imaging Data Analysis

The fMRI and anatomical data were pre-processed using SPM12^1^ based on Matlab. Preprocessing included the following steps: DICOM import, adduction of the center of anatomical and functional data to the anterior commissure, and reduction of the spatial distortion using the Field Map toolbox in SPM12 (Friston et al., 2007). Next, slice-timing correction for fMRI data, including the correction of hemodynamical response in space and time to avoid pronounced motion artifacts and head motion correction (Realign and Unwarp), were performed (Sladky et al., 2011). Anatomical data were segmented; both anatomical and functional data were normalized. Functional data were smoothed using a Gaussian function with a 6-mm isotropic kernel.

We used the SPM toolbox – WFU pickatlas^2^ to create a mask for the DMN. The mask contained the regions in the right and left hemisphere: intraparietal sulcus, Parahippocampal Gyrus, PCC, and MPFC (Jann et al., 2010; Di and Biswal, 2014). The ROIs for the DMN mask in the GLM analysis were created using Marsbar (toolbox for SPM).

The resting state was modeled using a General Linear Model with a discrete cosine basis set (GLM-DCT) consisting of 400 functions with frequencies characteristic to resting state dynamics of 0.0078–0.1 Hz (Biswal et al., 1995; Deco et al., 2011), as well as six nuisance regressors from each session capturing head motion, and the confound time-series from the extra-cerebral compartments. An F-contrast was specified across all frequencies of DCT, producing an SPM that identified regions exhibiting BOLD fluctuations within the frequency band. The preprocessing and conventional SPM analysis are the same as in our previous work dedicated to assess effective connectivity between four key DMN regions (Sharaev et al., 2016). For DCM analysis, the principal eigenvariate of a (8 mm radius) sphere was computed (adjusted for confounds) for each region and centered on the peak voxel of the aforementioned *F*-contrast.

**FIGURE 1:**
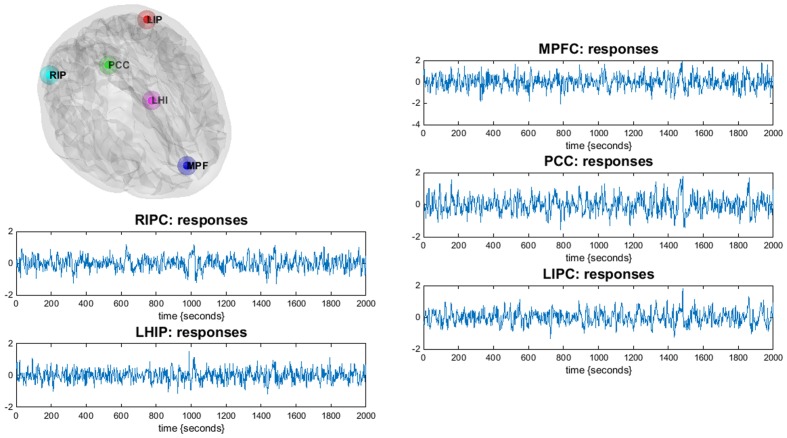
**Illustration of the DMN regions of the current study (only LHIP, not RHIP is shown): the DMN regions are identified using a conventional SPM analysis.** Corresponding time-series are principal eigenvariates of regions.

The obtained statistical parametric maps were then masked by a DMN mask based on previously reported MNI (Montreal Neurological Institute) coordinates for the DMN (Jann et al., 2010; Di and Biswal, 2014). For the extended model, we took as regions of interest (nodes) the most commonly reported four major parts of DMN: the mPFC [3, 54, -2], the PCC [0, -52, 26], left and right inferior parietal cortex LIPC [-50, -63, 32] and RIPC [48, -69, 35] (Di and Biswal, 2014), as well as two regions associated with hippocampus: left and right parahippocampal gyrus, LHIP [-22, -23, -14] and RHIP [19, -20, -10] (Jann et al., 2010). The square brackets contain the corresponding MNI coordinates of centers of regions (the principal eigenvariates of an 8-mm radius sphere was taken). The ROIs for DCM analysis were created in the DCM module of SPM. **Figure 1** illustrates the location of the main nodes of our analysis with the corresponding time series.

Functional connectivity was calculated in Conn (FC toolbox for SPM)^3^ based on the Fisher-transformed Pearson correlation coefficient. The significance level was set to *p* = 0.05 (FDR-corrected). Next, Fisher-transformed correlation coefficients between selected regions in the gray matter of human brain were calculated. We analyzed the same six ROIs of our DMN mask as in the DCM analysis – LHIP, LIPC, mPFC, PCC, RHIP, RIPC.

**FIGURE 2:**
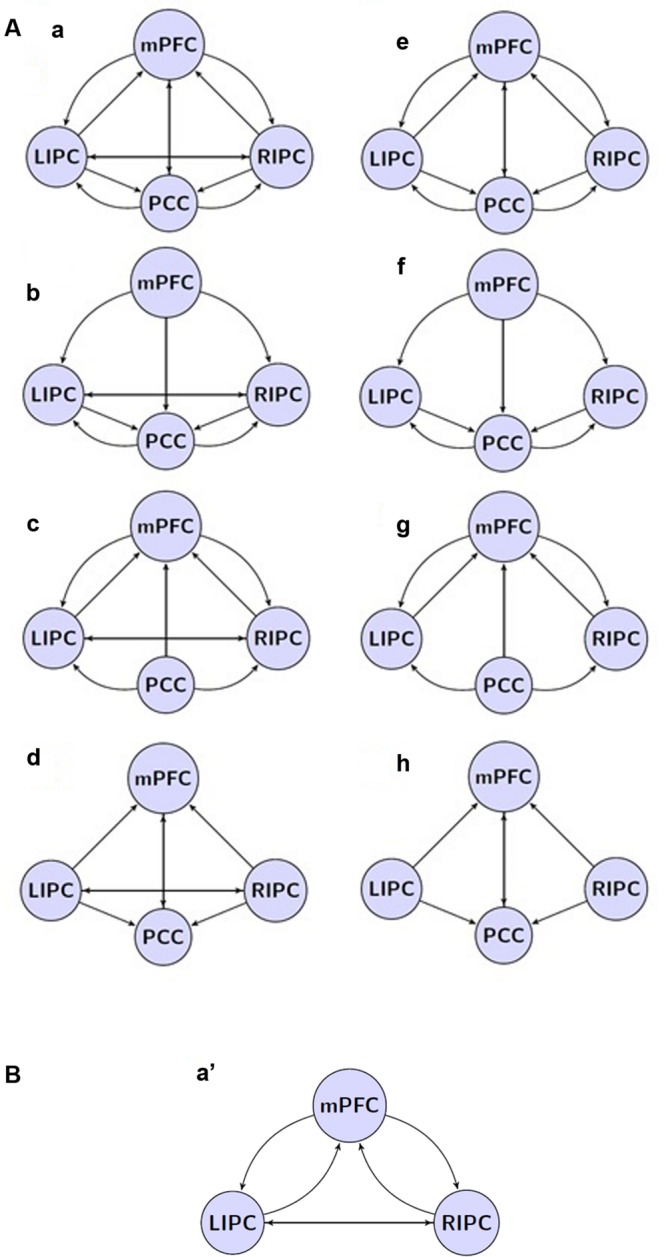
**The investigated model space.** **(A)** 8 model families (a–h) based on different connections between four main DMN regions. Double arrow means reciprocal connections. **(B)** a’ connectivity pattern: PCC region is removed, all other connections and regions are present.

According to the purpose of the study, we took as a basic model set eight models with four sources from our previous work (Sharaev et al., 2016): a full connected model, three models where different regions predominantly affected the other ones (mPFC, PCC, and bilateral modulation), and the same models but without direct connections between bilateral LIPC and RIPC, comprising eight model families, see **Figure 2A**.

**FIGURE 3:**
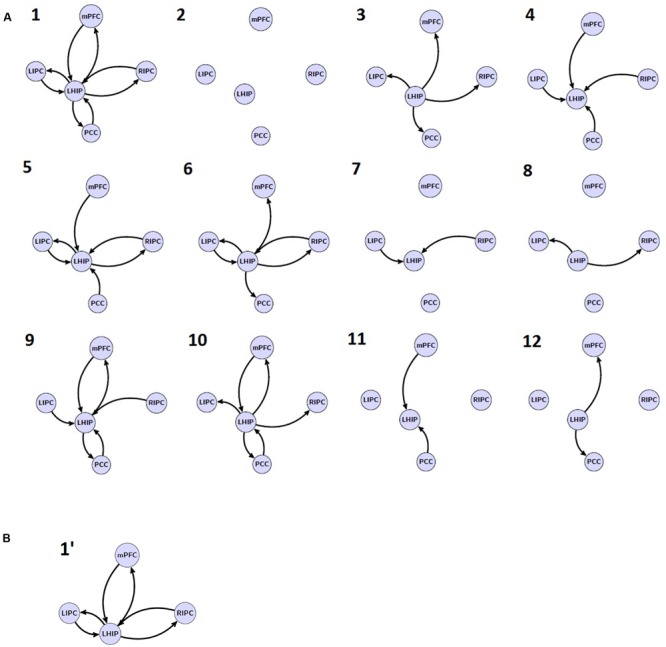
**The investigated model space.** **(A)** 12 model families (1–12) based on different LHIP/RHIP inclusion. **(B)** 1′ LHIP inclusion pattern without basic PCC region.

In the next step, one new region of interest, e.g., LHIP, was added to the basic set in different ways. Considering possible interactions, we firstly assumed a full connected (pattern 1) and a full disconnected (pattern 2) inclusion where an additional source either had reciprocal connections to all basic sources or had no connections to the basic set at all. We furthermore assumed two connection patterns where the additional source either only created outputs (drives) to the basic sources (pattern 3) or received input from them (pattern 4). We also assumed the following connectivity patterns: LHIP receives input from all basic sources and produces outputs for LIPC/RIPC (pattern 5), a symmetrical pattern where LHIP receives input from LIPC/RIPC and produces outputs for all four basic sources (pattern 6), a scheme where LHIP receives input from LIPC/RIPC producing no output (pattern 7), and where LHIP produces output for LIPC/RIPC but receives no input (pattern 8). Finally, we considered the same four connectivity patterns as (pattern 5) to (pattern 8) but where bilateral LIPC/RIPC were replaced with mPFC and PCC, respectively (9–12). Thus, we cover the majority of possible information flow patterns between the four key DMN regions and an additional region. This results in 12 model families (see **Figure 3A**); multiplying 12 by 8 basic families we obtain 96 models per subject. The same set of models is considered for the case of RHIP inclusion.

The second objective is to evaluate the model stability when removing one key region of interest: does the connectivity pattern between new sources change when removing a basic source? If not, then it would be a good verification of model: information flows are stable between analyzed regions, and if one of them is removed, the flows between other sources do not change significantly. For this purpose, we removed the PCC region from the extended model with LHIP inclusion so that only six basic connection patterns were possible (a′, e′, b′, f′, d′, h′, see **Figure 2B**, for example) and 12 LHIP inclusion patterns (all without the PCC region). This results in 12 additional families (see **Figure 3B** for an example of how to construct 1′ model); multiplying by 6 basic we obtain 72 models per subject.

Spectral DCM estimates the effective connectivity between neuronal populations, reflecting the observed (i.e., calculated) functional connectivity pattern in the frequency domain. This type of DCM uses a biologically plausible model of the dynamics of neuronal populations (including their causal relations) to generate predicted cross spectra and then compares it to the cross spectra among measured responses (Razi et al., 2015). For each subject, the schemes with no exogenous inputs were inverted using spectral DCM. Under the assumption that all participants used the same model, Fixed Effects (FFX) BMS (Stephan et al., 2009) was applied to determine the best model which balances the fit of data and the model complexity. Given the best model, the connectivity parameters from each subject were analyzed quantitatively using Bayesian Model Averaging (BMA) (Penny et al., 2010), to see whether some of them are stable across subjects. BMA was conducted to get the probability-weighted values of the model parameters. Bayesian Parameter Averaging (Penny et al., 2010) was also performed on the winning model.

## Results

Firstly, it should be noted that after performing conventional SPM analysis we found that 4 of 30 subjects did not reveal significant activity in either the LHIP or RHIP region. Obviously, such cases need to be more deeply investigated, but for the current study, the data from these subjects were excluded from the modeling step and further consideration.

**FIGURE 4:**
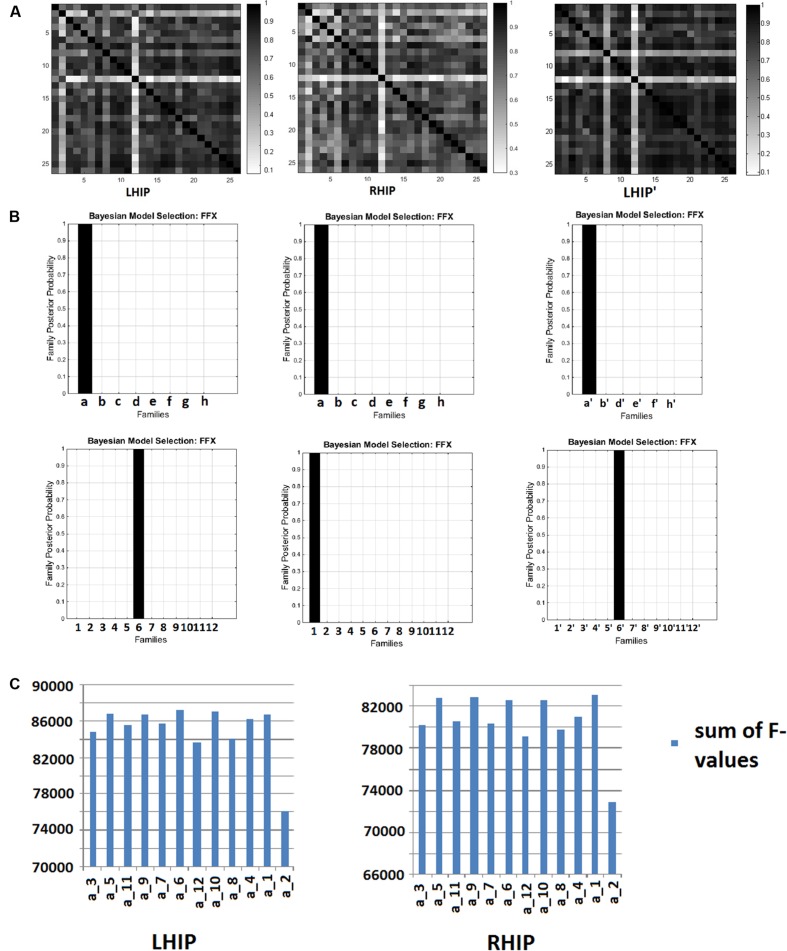
**Bayesian model selection (BMS) results.** **(A)** *F*-values correlation matrices across 26 subjects. LHIP – for 96 models with LHIP inclusion, RHIP - for 96 models with LHIP inclusion, LHIP’ - for 72 models with LHIP inclusion and PCC exclusion. **(B)** Model families posterior probabilities: for LHIP inclusion the winning families are **a** and **6**, for RHIP inclusion the winning families are **a** and **1**, for LHIP inclusion and PCC exclusion – **a′** and **6′**. **(C)** Sums of *F*-values across 26 subjects for winning family **a**. Models a_5, a_9, a_6, a_10, a_1 are performing better than others both for LHIP and RHIP inclusion. Absolute winners are models, which combined each winning family: **a_6**, **a_1,** and **a**′**_6**′.

For extended models with five sources (including LHIP or RHIP), after inverting 96 DCMs for 26 subjects, we received 96 × 26 = 2496 *F*-values (the log-evidence approximation for each model for every subject) and for the reduced model (with LHIP but without PCC), after inverting 72 DCMs, we received 72 × 26 = 1872 *F*-values. With a large number of models (e.g., 96 or 72), a question arises: do these models behave alike across subjects? If they are stable, i.e., the same model behaves in a similar way when applied to different subject data, then one can expect that the model reflects some factual neural processes. Otherwise, when the model performs randomly across subjects, it probably does not describe the same underlying neural activity. To answer this question, we counted correlations between individual *F*-values for 96 (in the case of LHIP/RHIP) and 72 (in the case of the reduced model without PCC) models across all 26 subjects. This results in correlation matrices with 26 rows as shown in **Figure 4A**. The color encodes the pairwise correlation value. The posterior probabilities of model families are shown in **Figure 4B**, and the sums of the models’ *F*-values across subjects for the winning family **a** is shown in **Figure 4C**.

As can be seen from the matrices, for most subject pairs, the correlation is rather high (mean value about 0.7–0.8), except for a couple of subjects for whom correlation was somewhat less. This is true for all models sets. Thus, we can conclude that models are quite stable across the group, since the same model behaves in a similar way when applied to different subject’s data, producing highly correlated *F*-values. Because there are no negative values in correlation matrices, this means that no models perform in the opposite way across subjects.

The winning families are **a** and **6** for LHIP inclusion**, a** and **1** for RHIP inclusion (**Figure 4B**). Regarding family **a,** one may recall from **Figure 2** it is the full connected base, which was the best model when analyzing four source models (Sharaev et al., 2016). This means that no matter how the LHIP/RHIP region is included, the best connection pattern between these four nodes remains the same. This is a significant finding, because it means that connectivity between four basic DMN nodes is not corrupted by adding the fifth node. Next, the 12 best performing models from family **a** are shown as 12 peaks in **Figure 4C**.

From **Figure 4B** (family **a**) and **Figure 4C**, it is clear that five models (a_5, a_9, a_6, a_10, a_1) are better than others, both for the LHIP and RHIP inclusion scheme. Though other models perform significantly worse and can be easily discarded, it becomes hard to distinguish between these five leading models. The same situation remains if we consider the number of wins, i.e., how often each model was the best one among 96 competing models in the group. The results are provided in **Table 1** below:

**Table 1:** Models and their number of wins in LHIP/RHIP group.

Model	Number of wins	
	LHIP	RHIP
a_5	2	0
a_9	4	4
a_6	2	4
a_10	2	1
a_1	8	7

*Numbers in columns reflect the total number of wins in the group of subjects for a particular model either with LHIP or RHIP inclusion*.

In both groups, the model a_1 (full connected base and full connected LHIP/RHIP areas) wins by a narrow margin, though by the BMS results, this model is the best one only in the RHIP group; in the LHIP group, the best model is a_6. All five models from **Table 1** imply that both hippocampal regions have causal connections to the majority of four key DMN regions. They also have a very similar architecture, which could explain why it becomes so difficult to find the best one among them. The BMA/BPA results are shown in **Tables 2** and **3** for LHIP/RHIP inclusion, respectively. The BMA results from the previous four source analysis are shown in **Table 4**.

**Table 2:** Mean connection strengths (in Hz) from BMA/BPA, LHIP inclusion.

BMA/BPA	from mPFC	from PCC	from LIPC	from RIPC	from LHIP
to mPFC		**0.15^∗^/**-0.04^∗^	**0.20/0.21**	**0.18/**0.09^∗^	**0.16^∗^/0.11^∗^**
to PCC	**0.10^∗^/**0.02^∗^		**0.23/0.21**	**0.22/0.14^∗^**	**0.29/0.21**
to LIPC	-**0.18/**-0.06^∗^	0.02^∗^/-0.06		**0.22/0.19**	**0.28/**0.07^∗^
to RIPC	-0.08^∗^/-0.06^∗^	-0.01^∗^/-0.05^∗^	**0.21/**0.09^∗^		**0.30/**0.05^∗^
to LHIP	0.00^∗^/0.00^∗^	0.00^∗^/0.00^∗^	**0.11^∗^/**-0.06^∗^	0.02^∗^/-0.04^∗^	

*In rows there are source regions, in columns – target regions. Non-trivial significant (*p* < 0.05) connections are shown in bold. (^∗^) non-significant after Bonferroni correction. Self-connections omitted for simplicity. Non-significant in four sources model but significant in five sources model is highlighted*.

**Table 3:** Mean connection strengths (in Hz) from BMA/BPA, RHIP inclusion.

BMA/BPA	from mPFC	from PCC	from LIPC	from RIPC	from RHIP
to mPFC		**0.10^∗^/**-0.04^∗^	**0.19/**0.06^∗^	**0.26/0.11**^∗^	0.09^∗^/0.07^∗^
to PCC	**0.13^∗^/**0.07^∗^		**0.27/0.30**	**0.35/0.21**	0.08^∗^/0.03^∗^
to LIPC	0.01^∗^/-0.01^∗^	0.00^∗^/-0.06^∗^		**0.27/0.19**	-0.08^∗^/-0.06^∗^
to RIPC	0.03^∗^/-0.03^∗^	-0.05^∗^/-0.05^∗^	**0.28/0.20**		-0.08^∗^/-0.07^∗^
to RHIP	0.03^∗^/0.02^∗^	-0.03^∗^/-0.04^∗^	**0.11^∗^/0.11^∗^**	**0.23/0.20**	

*In rows there are source regions, in columns – target regions. Non-trivial significant (*p* < 0.05) connections are shown in bold (^∗^) non-significant after Bonferroni correction. Self-connections omitted for simplicity*.

**Table 4:** Mean connection strengths (in Hz) from four source BMA.

BMA	from mPFC	from PCC	from LIPC	from RIPC
to mPFC		**0.15^∗^**	**0.28**	**0.32**
to PCC	**0.12^∗^**		**0.32**	**0.36**
to LIPC	-0.05^∗^	-0.01^∗^		**0.32**
to RIPC	-0.04^∗^	-0.05^∗^	**0.25**	

*In rows there are source regions, in columns – target regions. Non-trivial significant (*p* < 0.05) connections are shown in bold. (^∗^) non-significant after Bonferroni correction*.

**FIGURE 5:**
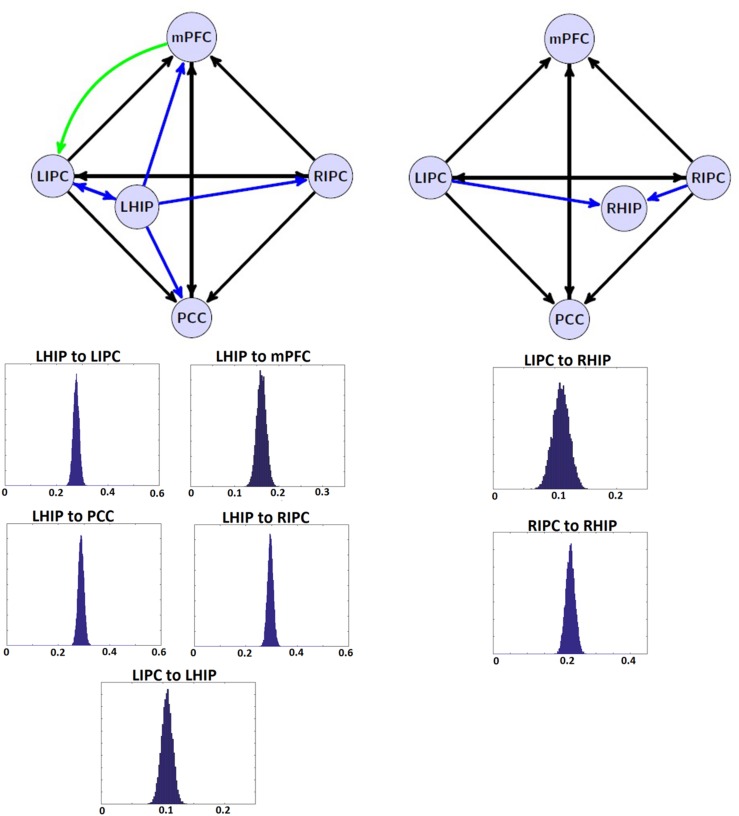
**The winning models at the group level and their non-trivial significant (*p* < 0.05) connections.** Left: the winning model for LHIP inclusion, green arrow depicts the new significant connection in comparison with four source modeling. Right: the winning model for RHIP inclusion. Blue arrows depict LHIP/RHIP effective connections with the base DMN sources. The BMA results are below the models for LHIP/RHIP inclusion, respectively. Only connections from/to LHIP/RHIP regions are represented in histograms.

The tables above show that in the base nodes set, only the connection from mPFC to LIPC significantly changed in the case of LHIP inclusion. This means that adding a new source (LHIP or RHIP) practically did not change the basic connectivity pattern. The best models with their BMA results are shown in **Figure 5** (note that BMA are plotted only for links from/to LHIP/RHIP).

Bayesian model selection on reduced models with LHIP but without PCC showed that the best model at the group level is a′_6′ – the same connectivity pattern as for the five source model including both LHIP and PCC. The number of wins by top performing models is: for a′_6′ and a′_1′ – both 5 wins; a′_5′ and d′_1′ – both 3 wins. There is a parity between a′_1′ and a′_6′ models, also a′_5′ and d′_1′ models are rather close to the winning ones. It is worth noting that a model with d′ base set outperforms many models with a full connected base set (a′). The most interesting part of the reduced models analysis is the BMA result in **Table 5**, comparing it to the five sources in LHIP BMA. Again, it can be seen that the majority of parameter estimates for the model with five sources and with four sources are very close, no new connections arose, and no connections disappeared. The significant difference (*p* < 0.05, insignificant after Bonferroni correction) between the parameters was found for only one connection: from LHIP to LIPC (0.28 versus 0.18, both positive – excitatory connections).

**Table 5:** Mean connection strengths (in Hz) from the reduced model BMA.

BMA	from mPFC	from LIPC	from RIPC	from LHIP
to mPFC		**0.32**	**0.29**	**0.23**
to LIPC	**-0.16**		**0.28**	**0.18^∗∗^**
to RIPC	-0.09^∗^	**0.19**		**0.27**
to LHIP	0.00^∗^	**0.15^∗^**	0.02^∗^	

*In rows there are source regions, in columns – target regions. Non-trivial significant (*p* < 0.05) connections are shown in bold. (^∗^) non-significant after Bonferroni correction. (^∗∗^) Parameters significantly different (*p* < 0.05) from five source scheme. Non-significant in four sources without LHIP model but significant in four sources without PCC model is highlighted*.

**FIGURE 6:**
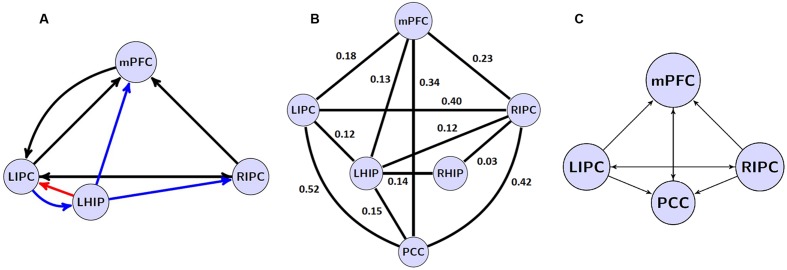
**(A)** the winning model at the group level without PCC region and its non-trivial significant (*p* < 0.05) connections. Blue arrows depict LHIP/RHIP effective connections with the base DMN sources, red arrow depicts the only connection, which parameter changed significantly (*p* < 0.05). **(B)** significant (*p* < 0.05) coefficients of functional connectivity between the six ROIs of the DMN. **(C)** The model with four basic ROIs taken from (Sharaev et al., 2016).

Overall, the important finding is that, with spectral DCM, there are stable patterns of effective connectivity even if a key region of the explored network is missed. The best reduced model at the group level is shown in **Figure 6A**.

A comparison of effective and functional connections is important as well. All significant functional connections are presented in **Table 6**; **Figure 6B**. The six regions of interest are interconnected with positive connections (correlations). The numbers are Fisher-transformed correlation coefficients. The model with four basic ROIs taken from (Sharaev et al., 2016) is shown in **Figure 6C**.

**Table 6:** Mean Fisher-transformed correlation coefficient values.

FC	mPFC	PCC	LIPC	RIPC	LHIP	RHIP
mPFC		**0.34**	**0.18**	**0.23**	**0.13**	0.02^∗^
PCC			**0.52**	**0.42**	**0.15**	0.03^∗^
LIPC				**0.40**	0.12	0.01^∗^
RIPC					0.12	0.03
LHIP						**0.14**

*Non-trivial significant (*p* < 0.05) connections are shown in bold. (^∗^) non-significant after FDR correction*.

**FIGURE 7:**
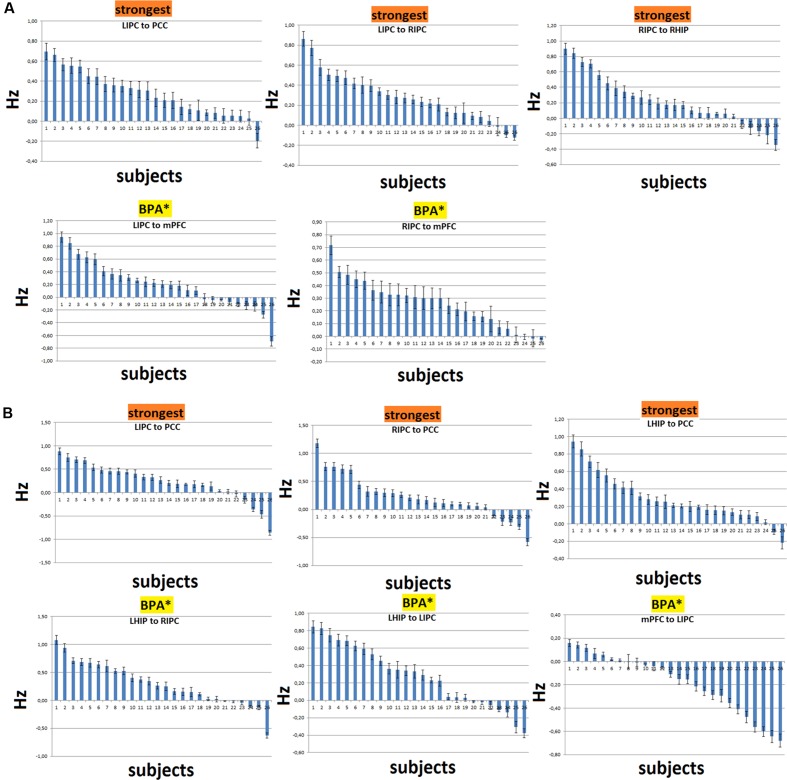
**Distributions of the connectivity parameters (in Hz) with their confidence intervals over subjects for the strongest connections and connections, significant after the BMA and non-significant after the BPA (shown as BPA^∗^).** **(A)** The case of RHIP inclusion. **(B)** The case of LHIP inclusion. The parameters are ranked in descending order. Here in most cases BPA results seem to be counterintuitive.

In order to assess the reproducibility of DCM parameter estimates, we examined the distributions of connection strengths together with their confidence intervals for the three strongest and most significant connections over subjects. For the model with RHIP inclusion, these are LIPC to PCC, LIPC to RIPC, and RIPC to RHIP. For the model with LHIP inclusion, these are LIPC to PCC, RIPC to PCC, and LHIP to PCC (**Figures 7A,B**, marked as “strongest”). We also examined connections which were strong and significant after the BMA and became non-significant after the BPA (**Figures 7A,B**, marked as “BPA^∗^”). It can be seen that there is a high degree of consistency over subjects for the strongest connections. Usually not more than 2–3 connections had the opposite sign. For the insignificant correlations? after the BPA connections, the picture is not so clear: for example, the LIPC to mPFC connection (**Figure 7A**) in 6 subjects is negative (with a big negative outlier), and the LHIP to LIPC connection (**Figure 7B**) is negative in 5 subjects. Despite this fact, the behavior of RIPC to mPFC (**Figure 7A**), LHIP to RIPC and mPFC to LIPC connections seems quite stable and determined over subjects, assuming that the BPA analysis produces here counter-intuitive results. Possibly, this is due to strong outliers in the group, or there could be other reasons. One of the reasons of such counter-intuitive behavior is described in (Kasess et al., 2010): BPA takes into account the posterior covariance structure and at high signal-to-noise ratios (SNRs), these covariances can have a profound impact on BPA results. They also say that “for SNRs greater than 2 the group estimate of connection strength provided by BPA increasingly underestimates the mean of the parameter distribution even though the single-subject estimates were quite accurate. Moreover, for SNRs >5, the average of the modulatory connection lies outside the actual range of the individual parameter estimates.” **Figure 7** shows that single-subject estimates are rather accurate. For all 26 subjects, in each voxel, we calculated the mean and the standard deviation of the corresponding time series to determine the SNR as in Welvaert and Rosseel (2013). The absolute voxel-wise minimum SNR value among all subjects was 2.4, ranging from this minimum to hundreds of units, which is consistent with resting-state time-series SNR (Welvaert and Rosseel, 2013). These findings, together with **Figures 7A,B**, lead us to the conclusion that BPA is not the best way to calculate parameter averages across subjects in our study. So, we preferred BMA (which is simply a weighted average) and performed further model analysis and discussion based on the BMA results.

The target regions in the current work were LHIP and RHIP. Our data on functional connectivity in part echoes that on causal connections showing that LHIP is more involved in the DMN than RHIP (see **Figure 5**). LHIP has connections with all key DMN regions. In contrast, RHIP has only two significant functional connections, whereby the stronger one is its connection to LHIP. Our functional connectome also includes links not reflected in the patterns of effective connectivity, e.g., a number of strong correlations of PCC with the activity of other key DMN nodes. Functional connections of PCC with LIPC and RIPC are stronger than LIPC and RIPS correlational links to hippocampal regions. Functional connections between RHIP and PCC, LIPC and RHIP, RHIP and mPFC are not significant.

Despite the fact that in the current work, the effective connectivity between two hippocampal regions was not directly assessed, one model combining the best LHIP and RHIP inclusion patterns (a_1 for RHIP and a_6 for LHIP with totally six nodes) was considered. Leaving the thorough analysis of both hippocampal regions inclusion for future research, we inverted only this particular model (without performing BMS), calculated parameter averages over subjects and directly compared LHIP and RHIP connections to the other four DMN nodes. It was found that: (a) LHIP/RHIP connections that were significant in the best 5-source model remain the same in 6-source model (except one RIPC to RHIP connection that fell slightly below the significance threshold), (b) connections from LHIP to mPFC, LIPC and RIPC are significantly (*p* < 0.01) stronger than the same connections from RHIP, (c) there is a very strong (0.44 Hz) and highly significant (*p* < 0.001) connection from LHIP to RHIP and no connection in the opposite direction. In our view, this preliminary analysis not only supports the overall picture of the discovered asymmetry, but also could be a good starting point to more exhaustive analysis of models with both hippocampal regions.

## Discussion

In this article, we extended our previous work on the causal relations within the DMN (Sharaev et al., 2016) by adding to its dynamic structure left and right parahippocampal regions. We also tested computational stability of our basic model by virtually removing one of the key DMN regions from the network. A discrete cosine basis set was used to model low frequency fluctuations in the brain, together with the spectral DCM to model the effective connectivity between the key DMN regions and the new target structures. The best models at the group level strongly suggested the same connectivity pattern between the four basic DMN regions – mPFC, PCC, LIPC, and RIPC, as revealed previously (Sharaev et al., 2016), and shed new light on the role of hippocampus in the resting state.

From the start of DMN studies, most researchers included HF in its composition (Greicius et al., 2004; Vincent et al., 2006). In addition, the DMN is often considered as a network implementing the basic state of consciousness (Gusnard and Raichle, 2001; Gusnard et al., 2001). In this role, it has to be closely related to HF which is crucial to basic cognitive functions of episodic memory (Tulving, 1985; Başar and Düzgün, 2016) and representation of surrounding space (Burgess et al., 2000; Moser and Moser, 2008). However, only a few reports addressed the lateralization of the DMN effective connections with respect to HF (Cui et al., 2015). By using spectral DCM as a tool, we for the first time found evidence for a strong asymmetric pattern in such relationships. LHIP demonstrated a high involvement in the DMN activity, with information outflow preponderant to all other DMN regions including RHIP, as shown by our preliminary analysis of 6-nodes interaction. Causal interactions of LHIP were bidirectional only in the case of LIPC. On the contrary, RHIP was mainly affected by inputs from LIPC and RIPC. In our view, this pattern of asymmetry in effective connections of the hippocampal regions may be related to lateralization phenomena in verbal and spatial domains documented in human neurophysiology, neuropsychology, and neurolinguistics (Howard and Templeton, 1966; Luria, 1966; Harrison, 2015). An obvious example of a drawback of such lateralized architecture is its potential vulnerability to unilateral destruction in functions of RHIP that could lead to the left-sided spatial hemi-neglect.

The authors of earlier studies often reported conflicting evidence on effective connectivity within the DMN (Jiao et al., 2011; Li et al., 2012; Di and Biswal, 2014). Models of effective connectivity from the present study are slightly different from the previous models and their power connections. For the first time, an inhibitory link was found between MPFC to LIPC (**Figures 5** and **6**), which may indicate the effort of the subjects to maintain resting state. This conclusion is supported by the data on dysfunction of the links between MFC and LIPC regions in patients with ADHD (Franzen et al., 2013). According to our results, bidirectional links between MPFC and PCC are relatively weak and statistically insignificant (**Figure 5**). Besides, our best reduced model remains stable even without the PCC node, when the link between mPFC and PCC is missing (**Figure 6**). The results are not consistent with the well-known strength of the structural links between the MPFC and PCC (Khalsa et al., 2014). We think that this is due to weakening effective connections of MPFC and PCC at rest, as were previously shown by Li et al. (2012) and Razi et al. (2015), although there are alternative data (Bajaj et al., 2013). Our conclusion can be supported by the data on the weakening of connections between functional networks in the resting state and their strengthening in the task performance (Di et al., 2013). On the other hand, our functional connectome provides a good average relatedness of these sites (**Figure 6B**), indicating a possible instability of the causal relationships in models which may indicate the effort with strong connections between MPFC and PCC.

Our extended models demonstrate the strong influence of both multimodal IPC zones on other DMN nodes (**Figure 5**), confirming previous reports (Jiao et al., 2011; Di and Biswal, 2014; Sharaev et al., 2016). Reduced models in their structure preserves these multimodal units and the majority of their bonds (**Figure 6A**). LIPC and RIPC influence MPFC and PCC, as well as interact with the hippocampal regions, doing this, as stressed above, in an asymmetric manner. The functional connectome also identifies asymmetric interactions with LIPC, RIPC and the hippocampal regions. LIPC interacts with LHIP more intensively than with RIPC with RHIP. At the same time, RIPC strongly interacts with LHIP than with RHIP (**Figure 6B**). The best reduced DCM model retains only the left hippocampal region, which affects all nodes of the reduced connectome and receives feedback from LIPC (**Figure 6A**). Functional connectivity confirms weak links of the right hippocampal region to other network nodes, except symmetrical structure of IPC. Some deviation in patterns of functional and effective connectivity is not surprising because of the large difference in temporal resolution of respective methods and a correlational character of the functional links.

It has to be noted that structural studies also demonstrate asymmetry in hippocampal regions such as differences in volumes (Pedraza et al., 2004; Shi et al., 2009). However, being dependent on a number of factors including handedness, age and anatomical position along the long axis (Lazić et al., 2015; Robinson et al., 2016), these differences seem to be less systematic than differences in functional activity. In addition, there is a kind of dissociation between the size of the hippocampus and its functional activity because LHIP, which functionally is more active, often has smaller volume than RHIP (Shi et al., 2009). One explanation for this discrepancy is that the increased activity of the left hippocampus in healthy subjects can lead to hippocampal volume reduction on the same side due to physiological pruning (Lazić et al., 2015; Okada et al., 2016). Functional lateralization seems to be a cause and not consequence of the anatomical difference. Our DCM data is consistent with the prominence of the LHIP functional activity. Of importance here is the known fact that the left parahippocampal region is primarily associated with verbal functions (Bernier et al., 2016), while the right one is involved in visual memory processes (Barnett et al., 2015). We are therefore inclined to regard the functional and effective lateralization of HF with the role of verbal memory and inner speech processes in the social meditation. The latter may be the very essence of the DMN activity as a whole (Li et al., 2014; Xie et al., 2016).

In general, our experimental DMN architecture can be interpreted as an optimally tuned system of operational rest (Bernhofer, 2016). It is characterized by a division of labor between left and right parahippocampal regions. RHIP plays a more receptive role by receiving streams of information from both IPCs and by building on the basis of this multimodal sensory input a bilateral representation of space. This spatial representation role seems to be restricted in the case of LHIP to representation of only the contralateral hemi-space. Having access to verbal episodic memory, LHIP takes over the function of the main driving force of activation within the DMN. In particular, it influences the decision-making center in MPFC (Lee and Seo, 2016). The latter is configured to behavioral rest and therefore inhibits sensory-motor structures in the leading hemisphere, i.e., LIPC (see green arrow in **Figure 5** left). As our model summarizes the data of a relatively large group of healthy subjects, it does not include the individual and temporal characteristics that must be investigated further.

Our study design is not without limitations. Due to the number of models for the simultaneously considered regions, we were limited by the causal analysis to not more than five nodes at once. For this reason, the present study left some of the obvious candidate connections without an in-depth analysis. In addition, the posterior hub of DMN, PCC, happened to play a strictly receptive role, not being as active in influencing other regions, as expected initially. This could be partially reflected in the results of our extraction procedure (see **Figure 6A**). Finally, it would be interesting to find functional and causal relations between DMN and the task-positive network (i.e., elucidating negative DMN connectivity - see Di Perri et al., 2016). This would be another obvious objective of such type of analysis. Similarly, to a study of individual and situational DMN properties, however, research related to task performances and goal-directed behavior would need the application of a larger battery of methods combining slow and fast neuroimaging approaches. Another potentially questionable point is our choice of averaging method – BMA instead of BPA. Despite the fact that BPA takes into consideration parameters (co) variance structure and thus more information about the estimate precision and parameter interdependence, BPA seems to be less applicable when examining systems with high SNR (>5).

As mentioned above, this work has two objectives. Besides investigating the HF place within the effective connections of the key DMN regions, we tested the stability of our models when adding or removing regions of interest. In the end, we could emphasize the finding that adding additional sources (LHIP or RHIP), as well as removing one of the regions (PCC in this case), does not change dramatically the overall connectivity pattern. Even if one takes into account several limitations of the study, this computational stability is surprising. It shows the potential of the spectral DCM as a reliable tool for neurocognitive research in health and in clinical conditions. Our main conclusion is therefore that the method can be of importance for constructing complex causal hypotheses (models) from simple connectivity circuits. In a similar vein, possibly, sophisticated psychological effects could be decomposed and evaluated in a parametric way.

## Author Contributions

VU: Work concept, article text (introduction, results, discussion), final article approval, accountable for all aspects of the work. MS: Mathematical methods, programming, data analysis, modeling, article text (introduction, methods, results) and pictures preparation, accountable for all aspects of the work. VZ: Experimental design, data acquisition and preprocessing, final article approval, accountable for all aspects of the work. SK: Data acquisition, article text (introduction), final article approval, accountable for all aspects of the work. VV: Experimental design, article text (introduction, discussion), final article approval, accountable for all aspects of the work. BV: Overall guidance, project idea, article text (introduction, neuropsychological aspects of discussion), final article approval, accountable for all aspects of the work.

## Conflict of Interest Statement

The authors declare that the research was conducted in the absence of any commercial or financial relationships that could be construed as a potential conflict of interest.

## Footnotes

1 http://www.fil.ion.ucl.ac.uk/spm/software/spm12/

2 http://uvasocialneuroscience.com/doku.php?id=uva_socia:wfu_pickatlas

3 http://www.nitrc.org/projects/conn
